# Case Report: Integrated imaging and molecular diagnostic approach to congenital hip dysplasia in a Hanwoo calf

**DOI:** 10.3389/fvets.2026.1788631

**Published:** 2026-04-29

**Authors:** Jiyeon Kim, Woojae Choi, Kyunghyun Min, Seri Hong, Sooyoung Choi, Danil Kim, Younghye Ro

**Affiliations:** 1 Department of Farm Animal Medicine, Seoul National University, Seoul, Republic of Korea; 2 Farm Animal Clinical Training Research Center, Institute of Green-Bio Science and Technology, Seoul National University, Pyeongchang, Republic of Korea; 3 Department of Large Animal Medicine, Gyeongsang National University, Jinju, Republic of Korea; 4 Minkyunghyun Animal Hospital, Hoengseong, Republic of Korea; 5 Department of Veterinary Diagnostic Imaging, Kangwon National University, Chuncheon, Republic of Korea; 6 Department of Large Animal Medicine, Kangwon National University, Chuncheon, Republic of Korea

**Keywords:** computed tomography, congenital disorder, Hanwoo, hip dysplasia, lameness

## Abstract

Congenital musculoskeletal disorders in calves, including those of genetic, infectious, or nutritional origin, can result in significant economic losses because of lameness, increased treatment costs, and reduced productivity. However, an accurate diagnosis is often difficult under field conditions. This report presents a case of bilateral hip dysplasia in a 1-month-old Hanwoo calf born prematurely that exhibited progressive lameness and joint deformities. Radiographic and computed tomography examinations revealed shallow acetabular sockets and underdeveloped femoral heads, consistent with congenital hip dysplasia. Additional abnormalities were observed in the left talus and calcaneus. Hematological and biochemical analyses results were unremarkable. Polymerase chain reaction assays for commonly implicated congenital viral pathogens yielded negative results; therefore, the underlying etiology was suspected to arise from genetic defects. The calf’s clinical condition deteriorated over time, and cessation of treatment was decided. Necropsy confirmed severe bilateral joint dysplasia, luxation, and femoral head erosion. This is the first documented case of bilateral hip dysplasia in a Hanwoo calf confirmed by advanced imaging and necropsy. This report emphasizes the diagnostic value and clinical significance of advanced diagnostic modalities in assessing comparable defects in Hanwoo cattle.

## 1 Introduction

In the cattle industry, where economic efficiency is a major determinant of value, diseases resulting in increased medical expenses and premature culling pose a critical threat to overall farm productivity (1–4). Genetic disorders are particularly problematic because their occurrence is difficult to predict or prevent and they frequently manifest during the neonatal stage (5–8).

Musculoskeletal disorders and lameness in calves arise from either congenital or acquired causes (5, 6, 9–11). Acquired causes include injuries from housing structures, other animals, or trauma from the dam, whereas congenital causes involve genetic abnormalities, maternal malnutrition, or in utero infections (5, 6, 11). Based on the authors’ clinical experience in the Republic of Korea, congenital abnormalities, such as atresia ani and contracted flexor tendons, are observed more frequently in native Korean cattle (Hanwoo) than in Holstein dairy cattle, which have undergone long-term selective breeding.

Congenital musculoskeletal disorders of infectious origin are most commonly associated with bovine viral diarrhea virus (BVDV), Akabane, Aino, Chuzan, and bluetongue viruses (1, 7, 11–15). BVDV can cause diarrhea, vesicular oral and nasal lesions, and musculoskeletal malformations such as perosomus elumbis (7). The Akabane virus, which is transmitted by *Culicoides* species, produces outcomes that vary with gestational stage, ranging from abortion to encephalitis, polymyositis, or arthrogryposis in calves (11, 13). The Aino and bluetongue viruses have also been implicated in congenital skeletal deformities (14, 15). Non-infectious etiologies include heritable factors, nutritional deficiencies, teratogenic drug or chemical exposure, and environmental influences that may lead to conditions such as arthrogryposis, muscle atrophy, or hyperflexible limbs (7).

While congenital malformations are clinically relevant in cattle, the application of advanced diagnostic methods, including imaging and laboratory analyses, is often limited in field practice due to cost and accessibility constraints (1, 10, 16–18). Therefore, detailed and diagnostically confirmed reports of congenital musculoskeletal abnormalities in cattle, particularly those based on multiple diagnostic modalities, remain limited. This may contribute to an incomplete understanding of their pathogenesis, etiological differentiation, and clinical significance, especially in native breeds such as Hanwoo cattle.

This report describes, to the authors’ knowledge, the first documented case of bilateral hip dysplasia in a Hanwoo calf, confirmed through a multimodal diagnostic approach involving clinical examination, diagnostic imaging, laboratory analyses, and necropsy. The findings provide clinical and gross pathological information and help expand the currently limited literature on such conditions in cattle.

## 2 Case description

### 2.1 History and Signalment

A 1-month-old male Hanwoo calf, born approximately 1 month prematurely, was referred to the Kangwon National University Animal Hospital (KNUAH). The primary clinical signs included abnormalities in the limbs and locomotion. According to the owner, the abnormalities were noted early but were initially considered a consequence of prematurity, and the calf was therefore observed without immediate intervention. As the condition failed to improve over time, veterinary evaluation was pursued. In the absence of diagnostic imaging facilities, the referring veterinarian conducted a physical examination and provisionally diagnosed forelimb arthrogryposis. Bilateral forelimb casting was applied and resulted in transient improvement; however, the clinical signs reappeared immediately after cast removal, leading to referral to the KNUAH for further assessment.

**Figure 1:**
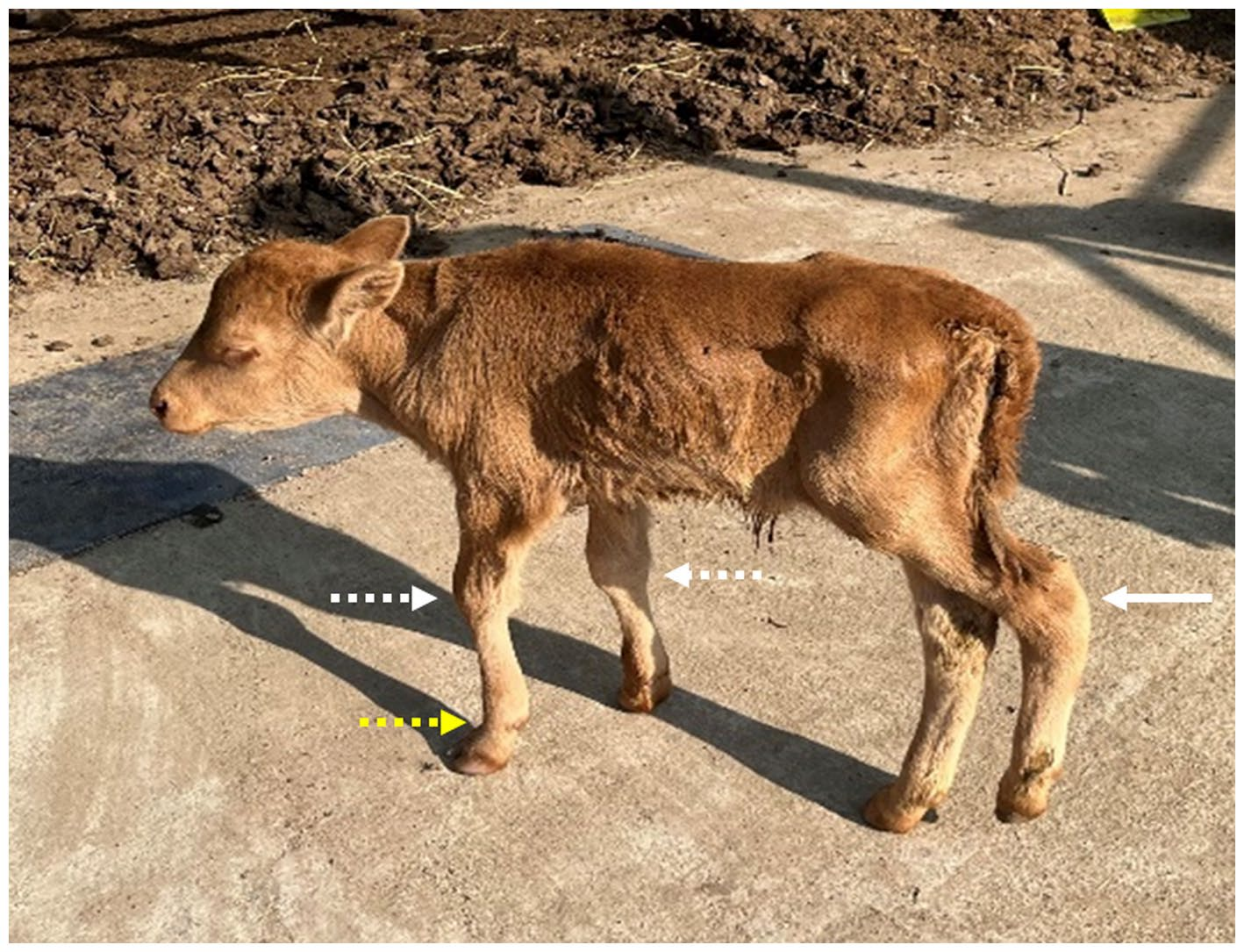
The standing posture of a 1-month-old Hanwoo calf exhibits abnormal flexion of the carpal joints suggestive of flexor tendon contracture, hyperextension of the forelimb fetlock joints (indicated by white and yellow dotted arrows, respectively), and abnormal angulation around the calcaneal region of the left hind limb (indicated by a white arrow).

At presentation to KNUAH, physical examination revealed abnormal posture and gait, without other significant clinical findings (Figure 1). The calf was unable to stand independently but could rise with assistance. Once standing, it showed an unstable gait, mainly associated with instability of the left hind limb and hip joint. Marked abnormal angulation of the calcaneus in the left hind limb was also observed. For safe handling and transport, xylazine hydrochloride (Rompun®, Elanco, Greenfield, IN, USA) was administered intravenously at a dose of 0.1 mg/kg to minimize the risk of injury.

### 2.2 Diagnostic imaging

**Figure 2:**
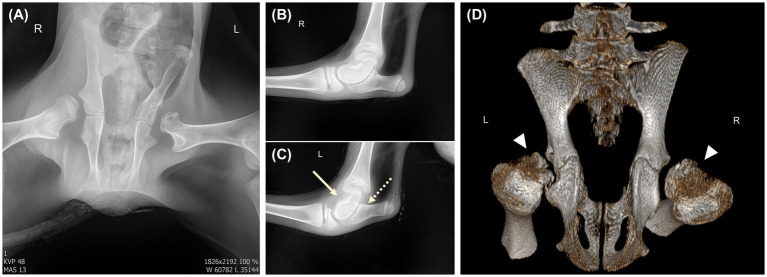
Radiographic images from the first referral. **(A)** X-ray of the pelvic region shows bilateral femoral head dysplasia and shallow acetabula (white arrowhead) in ventrodorsal view. **(B)** X-ray of the right hind limb fetlock joint. **(C)** X-ray of the left hind limb fetlock joint. Bone abnormalities and arthritis involving the left hind limb joint are identified, including dysplasia of the talus (white arrow), synovial swelling, and abnormalities of the distal left calcaneus (white dotted arrow). **(D)** Computed tomography image reveals bilateral acetabular dysplasia with extremely shallow acetabular sockets and underdeveloped femoral heads (white arrowhead).

Radiography (VXR-9M, DRGEM, Gwangmyeong, Korea) and computed tomography (CT; Somatom Emotion 6, Siemens, Erlangen, Germany) were performed at KNUAH. Pelvic radiography was conducted using an exposure setting of 48 kVp and 13 mAs in the ventrodorsal view. For CT imaging, the scanning parameters were set as follows: 120 kVp, 280 mAs, with a slice thickness of 1 mm. Imaging was performed in a dedicated radiation-shielded room, with CT exposure settings automatically determined through a preliminary scan to optimize parameters according to the animal’s body thickness and tissue density. The calf remained sedated during both procedures. Imaging revealed dysplasia and deformities in both femoral heads, resulting in bilateral hip joint abnormalities. The lesions were most prominent in the left iliac wing and femoral head (Figure 2). To objectively evaluate the degree of coxofemoral subluxation and acetabular shallowing, indices conventionally utilized in small animal practice were applied. These findings from this case were compared with quantitative data previously established by the authors in an age-matched normal calf (19, 20). The femoral head coverage index, used to assess subluxation, was 0% for both the left and right hip joints in the affected calf, in contrast to the 40% observed bilaterally in normal individuals. Regarding acetabular morphology, the acetabular depth in this patient was 0.89 cm (left side) and 1.3 cm (right side), compared to the normal depth of 1.6 cm. Furthermore, the acetabular index (depth-to-diameter ratio), which reflects the severity of socket shallowing, was 18.2 and 26% for the left and right sides, respectively, whereas it was 30% bilaterally in normal calves. These quantitative findings were more pronounced on the left side, consistent with the severity of the clinical and radiological presentations. Dysplasia or malformation of the left talus was also identified along with calcaneus irregularities. In the left limb, based on the radiographic findings showing synovial swelling, arthritis was diagnosed.

### 2.3 Blood analyses

Considering the premature birth and possibility of congenital abnormalities, blood samples from both the calf and its dam were collected to assess infectious agents associated with such conditions. Samples for complete blood and molecular diagnostics were collected in ethylenediaminetetraacetic acid (EDTA) tubes (K2EDTA, BD Vacutainer, BD, Franklin Lakes, NJ, USA), whereas plasma chemistry samples were collected in lithium-heparinized tubes (BD Vacutainer). All the samples were transported under refrigerated conditions. Hematological (Scil Vet abc Plus, HORIBA, Kyoto, Japan) and biochemical analyses (Cobas C311, Roche, Indianapolis, IN, USA) identified variations in total protein, albumin, glucose, and alkaline phosphatase (ALP) levels (Supplementary Table 1). These findings were interpreted as reflecting normal age-related physiological processes, such as rumen maturation, parturition and growth-related changes (21–23). However, they were deemed unlikely to have contributed to the clinical abnormality observed in the calf. Regarding the dam, a slightly elevated ALP level was noted; however, such variations are often observed during the post-parturition and lactation periods (23). The dam’s ALP elevation was considered post-parturition-related physiological variation rather than a pathological finding.

For pathogen detection, EDTA blood samples were centrifuged at 1,300 rpm for 15 min, and a buffy coat was used for nucleic acid extraction. Polymerase chain reaction (PCR; C1000 Touch™, BIO-RAD, Hercules, CA, USA) assays were conducted to detect for Akabane, Aino, Chuzan, bovine ephemeral fever, Ibaraki, bluetongue viruses and BVDV, all of which are implicated in premature birth or congenital abnormalities (7, 13, 15, 24, 25). To ensure diagnostic reliability, both positive and negative controls were included in PCR assays. Given the history of prematurity and concurrent congenital skeletal defects, we evaluated the calf for pathogens associated with reproductive failures and malformations. Although prominent neurological signs, such as inability to stand or ataxia, were absent, the presence of calcaneal malformations in addition to the primary hip dysplasia prompted further investigation. Consequently, we conducted diagnostic testing for specific pathogens known to cause late-term abortion, premature birth, and congenital deformities, even in the absence of typical neurological manifestations. Details of the primers and protocols are presented in Supplementary Table 2. All PCR assays yielded negative results and infectious agents were not detected.

### 2.4 Prognosis

**Figure 3:**
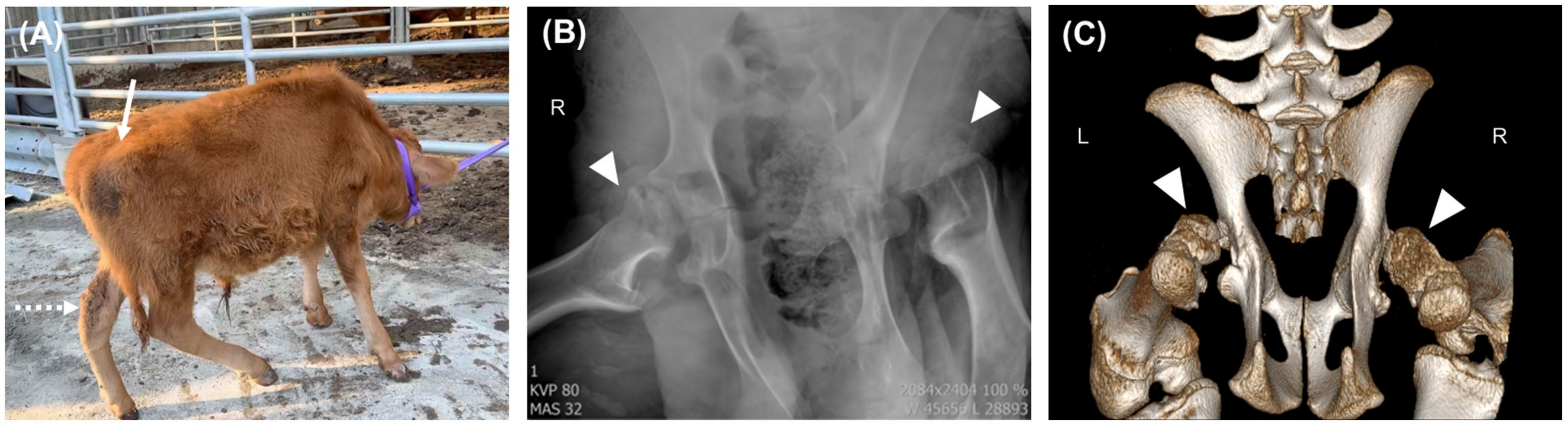
Standing posture and radiographic images at 6 months of age. **(A)** The calf’s standing posture has further deteriorated as a result of weight gain. Dorso-cranial luxation of the right hip joint (white arrow) and an abnormal angulation of the left calcaneus (white dotted arrow) are confirmed. **(B)** Ventrodorsal radiographic view of pelvis. X-ray images show bilateral osteolytic defects in the femoral heads and luxation between the femur head and acetabulum (white arrowhead). **(C)** Computed tomography image demonstrates dysplasia of both femoral heads (white arrowhead).

Following the initial diagnosis, the calf was returned to its dam at the owner’s request. During the subsequent 5 months, progressive deterioration in standing and ambulation was observed as its body weight increased exceeding approximately 80 kg (Figure 3A). Eventually, the owner elected to discontinue management. Additional diagnostic evaluations, including hematology, radiography, and CT, were performed to guide final decisions.

To summarize the clinical timeline, the calf was initially referred to the KNUAH at 1 month of age for a primary diagnostic evaluation. Five months later, at 6 months of age, the animal was re-referred at the owner’s request. Due to the lack of clinical improvement and the increasing difficulty of long-term management, the owner sought a definitive assessment regarding the feasibility of continued rearing and a more in-depth prognostic evaluation. Consequently, a comprehensive re-evaluation was performed to provide a final prognostic assessment to guide the owner’s decision on whether to continue rearing the calf. A detailed timeline of clinical events until euthanasia is illustrated in Supplementary Figure 1.

Imaging revealed severe acetabular dysplasia, shallow acetabular socket, and marked underdevelopment of the femoral head (Figures 3B,C). The femoral head was flattened and irregular with poor articulation to the acetabulum, resulting in luxation. Both the acetabulum and femoral head displayed sclerotic changes with evident subchondral bone erosion.

Based on these findings, the calf was diagnosed with multifocal osteodysplasia of the femoral head, which indicated a poor prognosis for recovery or productive performance. The calf was humanely euthanized following sedation and intravenous administration of T61 (4–6 mL/50 kg, MSD, Rahway, NJ, USA).

**Figure 4:**
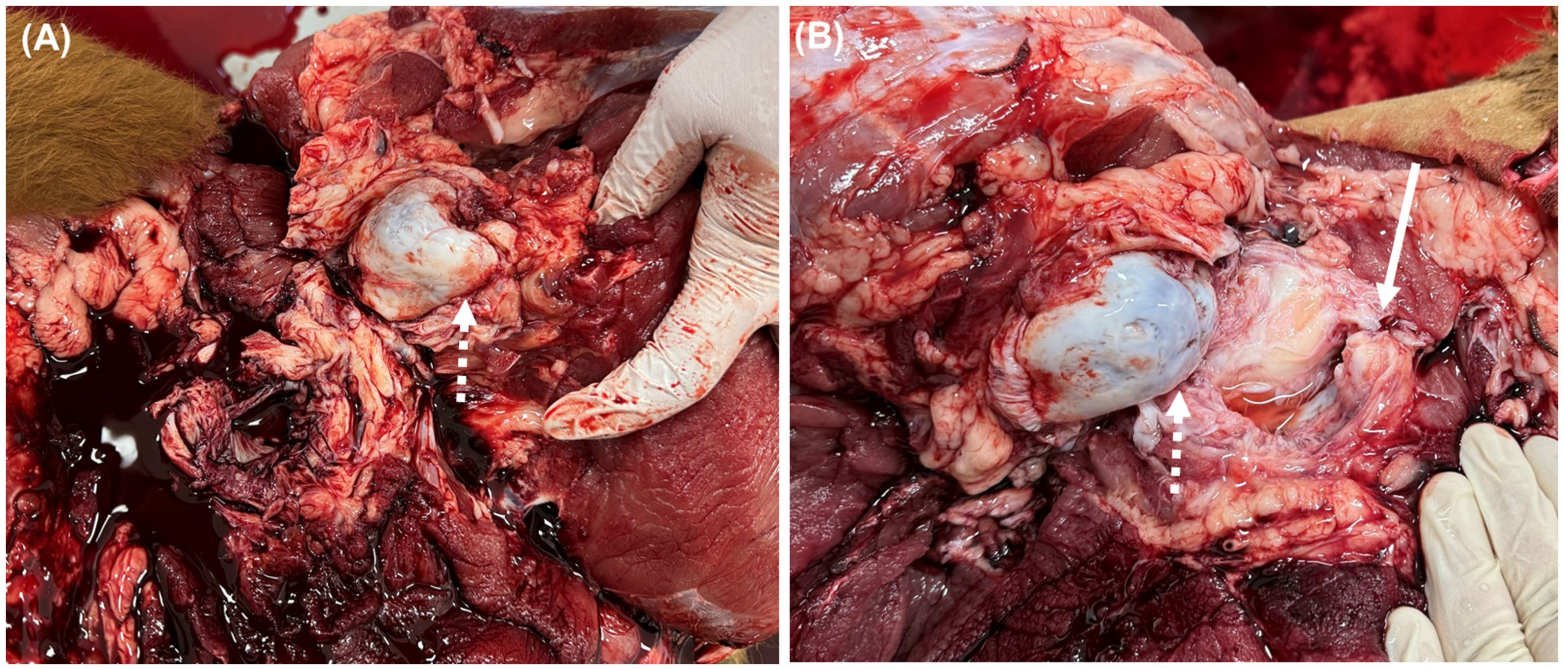
Post-mortem examination. **(A)** Bone erosion and dysplasia (white dotted arrow) in the left femoral head. **(B)** Dysplasia of the right femoral head (white dotted arrow) and shallow acetabulum socket (white arrow).

Necropsy was performed. Gross examination confirmed chronic hip luxation, absence or non-attachment of the round ligament, and extensive erosion of the femoral head. The acetabular socket was shallow with an irregular articular surface (Figure 4).

## 3 Discussion

As the underlying etiologies of congenital defects are often multifactorial, identifying the definitive cause of such abnormalities remains challenging. To differentiate these potential causes, a comprehensive evaluation including a detailed herd history and various clinical diagnostic tests are required. In the present case, no assisted parturition involving extraction forces was employed, and there were no clinical signs of trauma or falls during the perinatal period. Thus, external factors related to parturition were excluded. Nutritional and metabolic causes were deemed less likely in this case, as such conditions typically manifest as herd-level outbreaks and are associated with characteristic hematological changes. In the present case, biochemical parameters showed a tendency to normalize with age, whereas hematological values remained within reference ranges (Supplementary Table 1). Furthermore, the dam demonstrated an overall near-normal hematobiochemical profile. While the premature birth initially suggested infectious or genetic causes, the possibility of an infectious etiology was considered unlikely following negative results from extensive laboratory testing for various pathogens. Therefore, the disorder was presumed to be related to a genetic defect. Previous studies on congenital skeletal disorders have utilized whole-genome sequencing to identify gene defects (26, 27). While genomic approaches are gold standards for identifying mutations in genes such as Fibroblast Growth Factor Receptor 3 or Myosin Binding Protein C1 (26), genetic investigations were precluded in this study because viable samples were unavailable following the routine disposal of necropsy specimens.

Congenital bilateral hip dysplasia is characterized by an abnormally shallow acetabulum and lack of congruence between the acetabulum and femoral head (9). Hip dysplasia results in hip joint luxation, which, according to radiological findings, corresponds to subtype A of human hip dislocation classifications (9, 17, 28). While previous studies on hip dysplasia have included histopathological analyses (1), such examinations were not performed in the present case. Nevertheless, through the combined use of CT and radiography, we were able to characterize the femoral head dysplasia and predict that it was not associated with growth plate abnormalities. Furthermore, imaging allowed for the additional diagnosis of calcaneal malformation, extending beyond the hip joint involvement. Hip luxation is the most common orthopedic problem that affects the pelvic region in cattle and is difficult to diagnose and treat because of the well-developed muscle mass (10). Although hip dislocation in companion animals and humans is commonly diagnosed using radiography, CT, or magnetic resonance imaging, for large animals, such as cattle, determining a diagnosis is often restricted by cost and limited access to imaging facilities (1, 10, 16–18). According to Vlierbergen et al., ultrasonography revealed irregular cortical margins of the ilium in Belgian Blue cattle with hip dislocation; however, these findings were mainly observed in those with severe inflammatory changes (16, 17, 29, 30). In the present case, the primary pathology was congenital dysplasia rather than osteoarthritis. As there was no significant inflammation, ultrasonography was insufficient to determine a diagnosis; however, a definitive diagnosis was achieved using radiography and CT.

In this case, the primary goals of cattle production—such as breeding or fattening—were carefully considered in relation to the calf’s clinical condition. Considering the significant weight-bearing requirements of cattle compared to other animals, along with the rearing conditions of the farm, surgical intervention was deemed impractical. While surgical approaches, such as femoral head and neck ostectomy (FHNO), have been attempted in other species following a hip luxation diagnosis (31), they were not considered viable in this case due to the presence of multiple congenital malformations. While surgical interventions are routinely performed for other musculoskeletal disorders in cattle, they are generally not indicated for hip luxation (16, 30, 32, 33). In cattle with severe hip luxation, the economic impact regarding productivity often leads to culling as the preferred management strategy (16, 30, 34). The standard treatment for bovine hip dislocation is conservative management through closed reduction, with reported success rates ranging from 42 to 75% depending on various factors (16). An iliofemoral suture has been reported as a successful approach for treating hip dislocation in a calf that developed the condition following dystocia (16). In the present case, bilateral hip dysplasia, with acetabular dysplasia, was identified as the underlying cause of luxation. Even if FHNO had been attempted, dysplasia of the acetabulum would have limited the success of the surgical intervention, and the prognosis was expected to remain poor. The presence of additional musculoskeletal abnormalities in the left talus and associated joints suggested the presence of genetic defects. Consequently, after evaluating the impact on the animal’s future utility and welfare, humane euthanasia was selected over surgical treatment. This clinical decision was based on antemortem clinical findings and imaging assessments.

Previous studies have described unilateral hip dislocations in Simmental, Girolando, and Japanese Black calves (1, 16, 17). In agreement with the previously reported BVDV-negative Simmental case, no evidence of viral involvement was identified in the present case (1). The clinical significance of this report, however, lies in the bilateral hip dislocation, in contrast to the unilateral lesions described in the Simmental breed, and represents the first case in a Hanwoo calf.

Moreover, the application of CT helped to overcome the inherent limitations of two-dimensional radiography, enabling detailed three-dimensional visualization and quantitative evaluation of both subluxation severity and acetabular shallowing. Although such advanced imaging modalities offer substantial diagnostic advantages, their routine use in field settings remains constrained by economic considerations and the logistical challenges associated with transporting large animals. Nonetheless, these approaches may be particularly informative in animals with high genetic or economic value, such as breeding stock or high-yielding cattle. Additionally, standardized CT diagnostic criteria for the bovine hip joint remain limited. Therefore, the quantitative assessment presented in this report may serve as a preliminary reference for the development of diagnostic protocols in cattle with comparable musculoskeletal defects.

## Data Availability

The original contributions presented in the study are included in the article/Supplementary material, further inquiries can be directed to the corresponding authors.
