# Supplementary material for: Case Report: Integrated imaging and molecular diagnostic approach to congenital hip dysplasia in a Hanwoo calf
Source: Front Vet Sci. 2026 Apr 29;13:1788631. doi: 10.3389/fvets.2026.1788631 (PMC13170497; doi:10.3389/fvets.2026.1788631)
Supplement: Supplementary file 1 [file Data_Sheet_1.docx]

Supplementary Material

## Supplementary Figure 1. Timeline of clinical progression and management.

^*^ KNUAH, Kangwon National University Animal Hospital; m, months


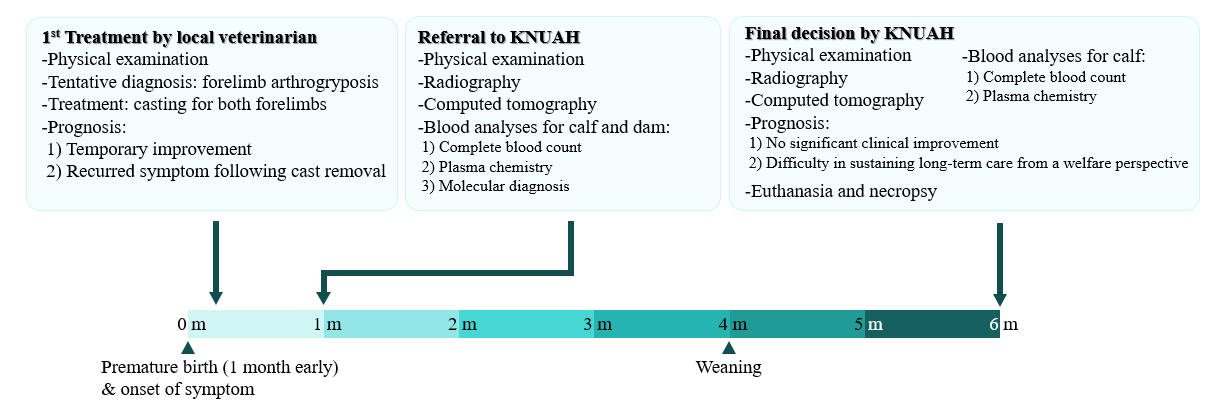


## Supplementary Table 1. Results of the complete blood count and plasma chemistry.

|  | Parameter (unit) | **Calf** | | **dam** | **Reference Range^**^** | | |
| --- | --- | --- | --- | --- | --- | --- | --- |
|  |  | Initial referral | Final decision^*^ | Initial referral |  |  |  |
| Complete Blood Count | White blood cell (10^3^/mm^3^) | 5.6 | 7.6 | 7.9 | 5.0 | - | 10.0 |
|  | Red blood cell (10^6^/mm^3^) | 9.71 | 10.16 | 8.67 | 5.5 | - | 11.0 |
|  | Hemoglobin (g/dL) | 11.3 | 10.7 | 14.5 | 9.0 | - | 18.0 |
|  | Hematocrit (%) | 35.8 | 31.9 | 44.5 | 30.0 | - | 50.0 |
| Plasma chemistry | Total Protein (g/dL) | 4.8 | 5.6 | 7.5 | 7.2 | - | 9.0 |
|  | Albumin (g/dL) | 2.2 | 3.0 | 3.5 | 3.2 | - | 4.2 |
|  | Glucose (mg/dL) | 134 | 69 | 80 | 31.0 | - | 77.0 |
|  | Alkaline Phosphatase (U/L) | 829 | 324 | 142 | 23.0 | - | 78.0 |
|  | Aspartate Aminotransferase (U/L) | 73 | 151 | 54 | 53.0 | - | 162.0 |
|  | Creatine Kinase (U/L) | 131 | 159 | 117 | 77.0 | - | 265.0 |
|  | Calcium (mg/dL) | 8.951 | 10.477 | 9.965 | 8.3 | - | 10.4 |
|  | Phosphate (mg/dL) | 6.556 | 8.153 | 7.071 | 4.2 | - | 7.7 |

^*^ Five months after the initial referral, when the owner decided to suspend the rearing.

^**^ Reference range for adult cattle.

## Supplementary Table 2. Primer sequences and product sizes for viruses associated with congenital disorders.

| **Virus** | **PCR type** | **Primer sequence (5’-3’)** | **Product size (bp)** | **Reference** |
| --- | --- | --- | --- | --- |
| Akabane virus | RT-PCR | [F] TAACTACGCATTGCAATGGC  [R] TAAGCTTAGATCTGGATACC | 709 | (11) |
|  | Nested PCR | [F] GAAGGCCAAGATGGTCTTAC  [R] GGCATCACAATTGTGGCAGC | 230 |  |
| Aino virus | RT-PCR | [F] CCCAACTCAATTTCGATACC  [R] TTTGGAACACCATACTGGGG | 649 |  |
|  | Nested PCR | [F] CCATCGTCTCTCAGGATATC  [R] ACAGCATTGAAGGCTGCACG | 345 |  |
| Chuzan virus | RT-PCR | [F] TGGCTTTCTGAGGCGTTTCAGA  [R] GGTTGCTCAATATGCCAAGCGA | 305 |  |
|  | Nested PCR | [F] GATGAAGCAGCGGATGTTTT  [R] AGCAGGTTAAGCCATACTCT | 220 |  |
| Bovine ephemeral fever virus | RT-PCR | [F] CCTACATTGAGAGTGCCACA  [R] CTTCCGTGAACAGCAACATC | 440 |  |
|  | Nested PCR | [F] TGAGAGTGCCACAAGGTAAA  [R] GCAGAGCGACCTAATCTATA | 328 |  |
| Ibaraki virus | RT-PCR | [F] CCTAGATGTTCAATAGCAAACCTAATT  [R] TAACATTTCGTTATAACAATAATAATT | 660 |  |
|  | Nested PCR | [F] ACGTGCTTACCAACAGGAGA  [R] TCAAGCTGTCGTGCCATATT | 314 |  |
| Bluetongue virus | RT-PCR | [F] GGAACAGGATATAATGGTTGGGIIIIIATTGATGTCG  [R] TCTCATTTCTRTGCGTAGGTTCAAIIIIICTGTTGAAAG | 440 | (19) |
| Bovine viral diarrhea virus | RT-PCR | [F] GTAGTCGTCAGTGGTTCG  [R] GCCATGTACAGCAGAGAT | 365 | (20) |

* RT-PCR, Reverse Transcription PCR; F, Forward; R, Reverse.
